# Invasion of the pterygoid plates: an indicator for regional lymph node failure in maxillary sinus cancer

**DOI:** 10.1186/s13014-020-01726-w

**Published:** 2021-01-06

**Authors:** Yasuo Kosugi, Terufumi Kawamoto, Masaki Oshima, Mitsuhisa Fujimaki, Shinichi Ohba, Fumihiko Matsumoto, Naoto Shikama, Keisuke Sasai

**Affiliations:** 1 Department of Radiation Oncology, Juntendo University, 2-1-1 Hongo, Bunkyo-ku, Tokyo, 113-8421 Japan; 2 Department of Otorhinolaryngology–Head and Neck Surgery, Juntendo University, Tokyo, Japan

**Keywords:** Maxillary sinus cancer, Lymph node metastases, Elective nodal irradiation, Intraarterial chemotherapy

## Abstract

**Background:**

The aim of this study was to evaluate the long-term treatment results of combined superselective intraarterial chemotherapy and radiation therapy for advanced maxillary sinus cancer (MSC) and the incidence of regional lymph node failure, and to reveal the clinical and anatomical predictive factors for metastasis.

**Methods:**

We retrospectively evaluated 55 consecutive patients with locally advanced squamous cell carcinoma of the maxillary sinus who were treated with external radiotherapy and superselective intraarterial chemotherapy. Elective nodal irradiation (ENI) was performed only in the clinical node-positive (cN+) cases and not in the clinical node-negative (cN0) cases. Results: Thirty-eight patients were cN0, and 17 were cN+ at diagnosis. Regional lymph node metastases occurred in 7 of 38 patients with cN0, and 2 of 17 with cN+ during the median follow-up period of 36 months. There were more cases of high-grade (3 or 4) late adverse events in the ENI group than in the non-ENI group (13% vs. 41%, respectively; *p* = 0.03). In cN0 cases without ENI, invasion of the pterygoid plates (57% vs. 90%; *p* < 0.01) and oral cavity (35% vs. 92%, with invasion vs without invasion, respectively; *p* = 0.02) was significantly correlated with a low 5-year regional recurrence-free rate.

**Conclusions:**

Patients with MCS and invasion of the pterygoid plates and oral cavity can be considered appropriate candidates for ENI.

## Introduction

Maxillary sinus cancer (MSC) is a relatively rare disease, with an incidence of 1 per 100,000 person-years, accounting for 3% of all head and neck cancers [1]. There is a paucity of prospective randomized studies to lead clinical practice because of the rarity of this disease. The combination of resection, external beam radiotherapy, and systemic chemotherapy is an international standard treatment [2, 3]. The National Comprehensive Cancer Network (NCCN) guidelines recommend elective nodal irradiation (ENI) for patients with T3 or T4 MSC, even in clinical node-negative (cN0) cases [2]. A meta-analysis indicated that ENI can significantly reduce the incidence of regional failure in cN0 cases. However, the use of ENI for MSC is controversial because of the variability in the frequency of regional failure of 3–33% [1] and because of the lack of reliable evidence. Furthermore, the long-term safety of ENI as a treatment for patients with advanced MSC is unknown because of the disease’s poor prognosis [3, 4].

The combination of radiation therapy and intraarterial (IA) chemotherapy is a promising treatment for unresectable or surgery-refused MSC because of its high local control rate and overall survival [5, 6]. This is a popular treatment in Japan, and although it is not a standard method, we have treated patients with locally advanced MSC using this combination, and reported good results [7]. In this setting, we have performed ENI only for patients with regional lymph node metastasis (cN+). The aim of the current study was to evaluate the long-term treatment results of advanced MSC and to clarify the incidence of regional lymph node failure and its predictive factors.

## Materials and methods

### Study design and data collection

We retrospectively analyzed data for 55 consecutive patients with locally advanced squamous cell carcinoma of the maxillary sinus who were treated with external beam radiotherapy and IA chemotherapy from April 2009 to August 2017 at our institution. All patients had unresectable MSC or refused surgery. The ethics committee of our hospital approved the study protocol (approval number: 19-173), and the study was conducted in accordance with the principles of the Declaration of Helsinki. In accordance with the Union for International Cancer Control (UICC, 7th edition) [8], staging was performed based on physical examination, computed tomography (CT), magnetic resonance imaging (MRI), and/or positron emission tomography-CT (PET-CT) findings.

### Treatment

External beam radiotherapy was provided using three-dimensional conformal radiotherapy (3DCRT) in 44 patients until June 2015 and intensity-modulated radiation therapy (IMRT) with helical tomotherapy in 11 patients after July 2015. The patients were immobilized using a thermoplastic mask, and radiotherapy and IA chemotherapy were performed simultaneously. The primary sites and lymph node (LN) metastases were irradiated with 60–70 Gy (median, 60 Gy) in 30–35 fractions over 6–7 weeks. The dose of ENI was 46 Gy in 23 fractions by 3DCRT or 50 Gy in 25 fractions by IMRT (median, 46 Gy) for cN+ cases; ENI was not performed in cN0 cases. Ipsilateral levels I, II, and III were set for regional irradiation, and contralateral levels II and III were additionally included in cases of bilateral LN metastases at presentation. We provided 3DCRT with 4- or 10-MV photons produced by the Clinac 21EX or TrueBeam (Varian Medical Systems, Palo Alto, CA) systems and IMRT with 6-MV photons produced by a TomoTherapy HD unit (Accuray Inc., Sunnyvale, CA).

Cisplatin at a dose of 150 mg/m^2^ was superselectively administered to mainly the maxillary artery and branches of the external carotid artery, which perfused the primary and lymph node lesions, and the procedures were repeated 4–5 times during treatment. During infusion of the agent, 200-fold sodium thiosulphate was additionally injected via a catheter in the brachiocephalic vein introduced via the subclavian vein to neutralize the adverse effects of the drug.

### Anatomical considerations

**Fig. 1:**
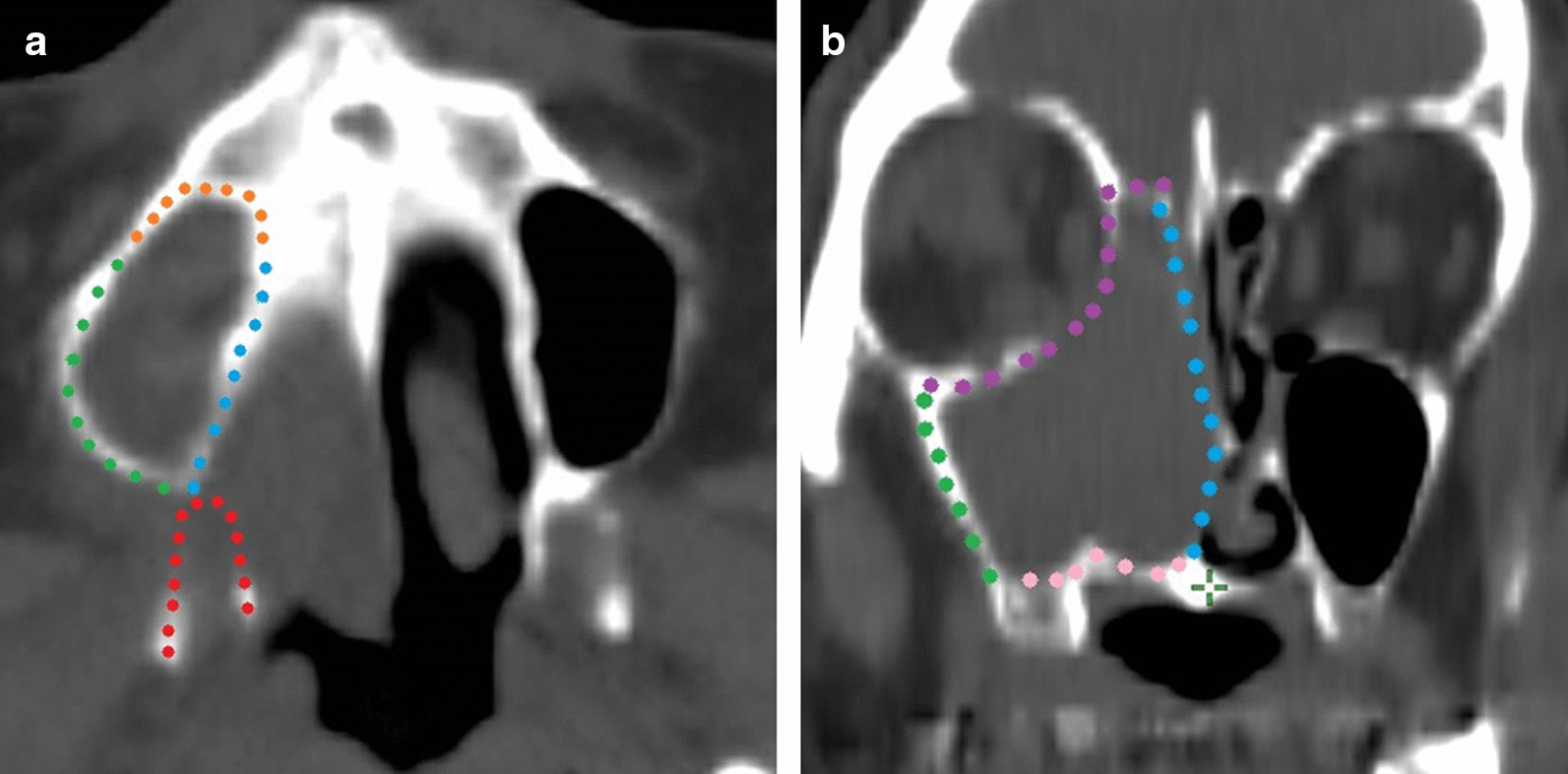
Anatomical direction of invasion of the primary lesion. Tumors with posterior (**a**) and cranial and medial extension (**b**) in the present study. Orange: anterior side; Red: posterior side; Blue: medial side; Green: lateral side; Purple: cranial side; and Pink: caudal side. + , combined posterior and lateral extension

Previous reports suggest that the anatomical sites of tumor invasion are particularly important regarding regional failure [9–11]. Therefore, we classified tumor invasion into six directions (Fig. 1) based on a study by Jeon et al. [11], with some modifications. The anterior, posterior, medial, lateral, cranial, and caudal extent were defined as the cheek, pterygoid plates, nasal cavity, masticator space, orbit or skull base, and oral cavity, respectively. Additionally, we analyzed the nasopharyngeal extent separately because other investigators reported that this is an important prognostic factor [9, 10]. Invasion was judged according to bone destruction on CT, and that into the nasopharynx was judged when the mass was clearly protruding into the nasopharyngeal cavity. Destruction of the posterior wall in a previous study was approximately equal to combined posterior and lateral extent in this study [11].

### Statistical analysis

Factors associated with regional failure, local failure, distant metastases, and overall survival (OS), namely invasion direction, sex, age, Eastern Cooperative Oncology Group performance status (PS), and T-stage, were analyzed. Pearson’s chi-squared test, Fisher’s exact test, the log-rank test, and Cox proportional- hazards models were used to analyze the correlation between the factors and regional failure or LN metastases at presentation. Regional control and survival outcomes were calculated using the Kaplan–Meier method. Adverse events (AEs) were assessed and documented according to the National Cancer Institute Common Terminology Criteria for Adverse Events version 4.0 [12]. AEs occurring within 3 months after treatment were defined as acute, and those occurring ≥ 3 months after treatment were defined as late. All statistical analyses were assessed at a significance level of 0.05 using JMP 12 (SAS Institute; Minato-ku, Tokyo, Japan).

## Results

### Patients’ characteristics and lymph node metastasis at presentation

**Fig. 2:**
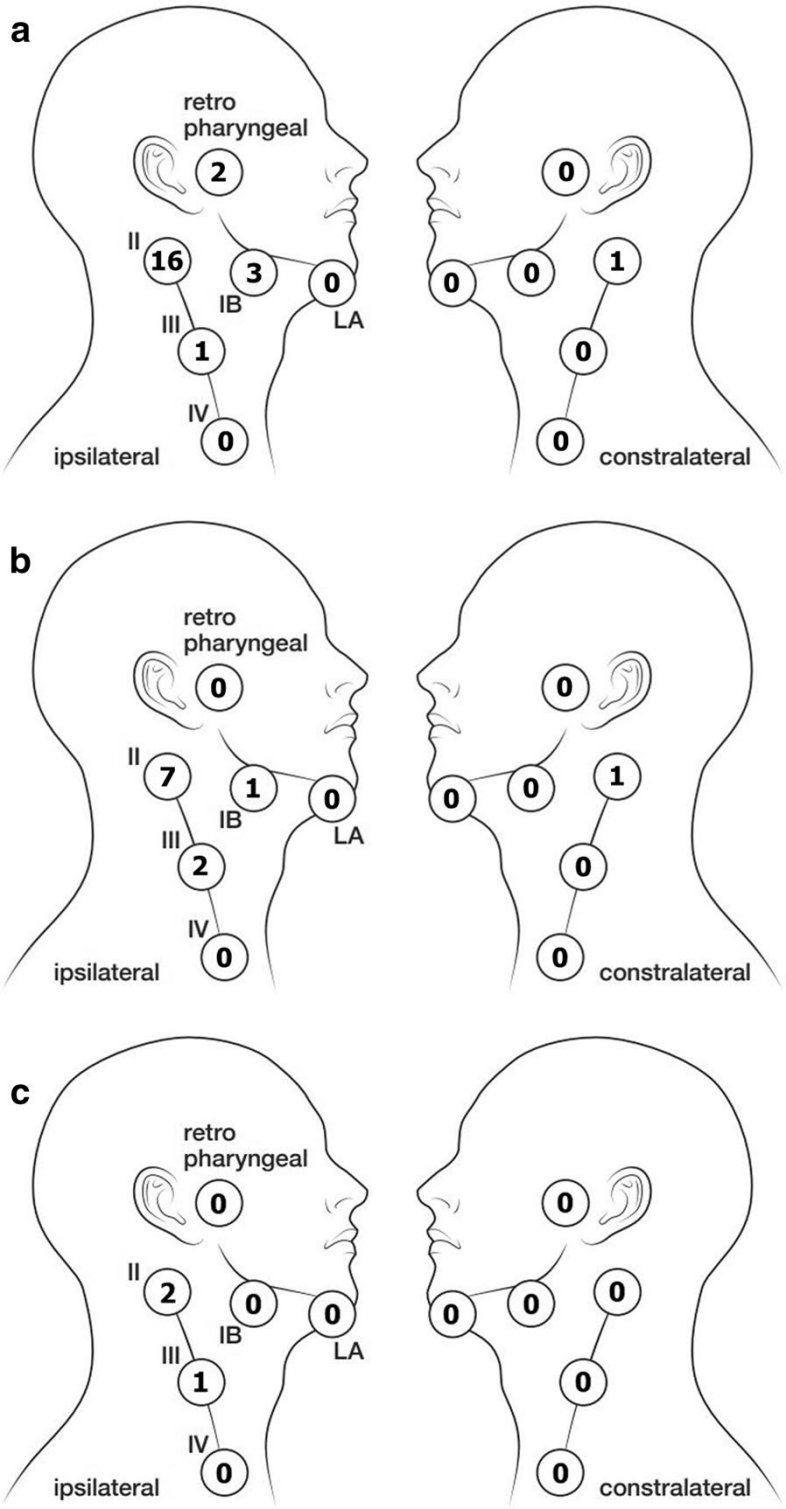
Number of cases of lymph node metastasis at each level at presentation (**a**) and regional failures in cN0 (**b**) and cN+ (**c**) cases

Table 1 presents the patients’ characteristics. The median age was 70 years (range, 38–91 years). For staging, CT was performed in all cases. MRI and PET-CT was performed in 31 and 13 out of 55 patients, respectively. Most patients had cT4 disease. In the 17 cN+ cases at diagnosis, ipsilateral levels I and II were the most prevalent regions of metastasis. Figure 2a shows the distribution of LN metastases at diagnosis.

**Table 1:** Patients’ characteristics (a), frequency of lymph node (LN) metastasis at presentation according to each characteristic (b), and the 5-year regional recurrence-free rate in cN0 cases by characteristic (c)

(a) Characteristic	(b) LN metastasis at presentasion (%)			(c) 5-year regional recurrence-free rate in cN0 cases (%)		
	No. (%)		*p* value	No		*p* value
Age (years), median (range)	70 (38–91)					
≥ 70 years	30 (55)	23	0.18	23	76	0.7
< 70 years	25 (45)	40		15	76	
Gender						
Male	46 (84)	30	1	32	81	0.44
Female	9 (16)	33		6	53	
ECOG PS						
0	42 (76)	31	0.82	29	78	0.26
1	11 (20)	27		8	57	
≥ 2	2 (4)	50		1	–	
T factor						
3	5 (9)	0	0.12	5	75	0.96
4a	26 (47)	42		15	63	
4b	24 (44)	25		18	82	
N factor						
0	38(69)					
1	5 (9)					
2b	11 (20)					
2c	1 (2)					
Imaging studies at presentation						
CT	55 (100)					
MRI	27 (49)					
PET‐CT	13 (24)					
Direction of tumor invasion medial site						
Yes	53 (96)	30	0.52	37	73	0.58
No	2 (4)	50		1	100	
Lateral site						
Yes	45 (82)	33	0.33	30	67	0.16
No	10 (18)	20		8	100	
Cranial site						
Yes	52 (95)	29	0.22	37	73	0.64
No	3 (5)	66		1	100	
Caudal site						
Yes	31 (56)	32	1	21	35	0.02
No	14 (44)	29		17	92	
Anterior site						
Yes	48 (87)	31	1	33	80	0.83
No	7 (13)	29		5	71	
Posterior site (pterygoid plates)						
Yes	34 (62)	41	0.02	20	57	< 0.01
No	21 (38)	14		18	90	
Nasopharynx						
Yes	5 (9)	60	0.17	2	100	0.53
No	50 (91)	27		36	72	
Posterior wall^+^						
Yes	50 (91)	34	0.14	33	69	0.3
No	5 (9)	0		5	100

### Regional failure

After a median follow-up period of 36 months, 24 patients died, 21 were alive, and 10 were lost to follow-up at 6, 10, 16, 17, 19, 31, 62, 65, 89, and 95 months, respectively. The median follow-up period for the 21 surviving and 10 censored patients was 63 months (range, 5–113 months). Of the 10 patients lost to follow-up, 4 developed disease progression and were considered dead as of the day of the last visit (at 6, 16, 19, and 65 months, respectively) in subsequent survival analyses. The analyses were censored as of the last visit day for six other patients without tumor recurrence. The 1-, 3-, and 5-year OS rates were 78%, 53%, and 50%, respectively, and there were 14 local failures, 9 regional, and 10 distant. The 5-year local control (LC) and distant metastatic recurrence-free survival (DMFS) rates were 67% and 47%, respectively. The 5-year OS, LC, and DMFS rates for all cases or cN0 cases for each patient characteristic are shown in Tables 2 and 3.

**Table 2:** The 5-year OS, LC, and DMFS rates for all patients for each patient characteristic

Characteristic		5-year OS rate (%)		5-year LC rate		5-year DMFS rate	
	No. (%)		*p* value		*p* value		*p* value
Age (years)							
≥ 70 years	30 (55)	36	0.03	57	0.45	51	0.21
< 70 years	25 (45)	60		78		44	
Gender							
Male	46 (84)	52	0.49	72	0.19	46	0.53
Female	9 (16)	42		42		50	
ECOG PS							
0	42 (76)	40	< 0.01	67	0.9	51	< 0.01
1	11 (20)	20		68		41	
≥ 2	2 (4)	–		100		–	
T factor							
3	5 (9)	60	0.83	80	0.5	80	0.83
4a	26 (47)	45		57		44	
4b	24 (44)	56		76		43	
N factor							
Negative	38 (69)	51	0.85	67	0.98	55	0.39
Positive	17 (31)	47		68		31	
Direction of tumor invasion medial site							
Yes	53 (96)	48	0.4	65	0.38	47	0.67
No	2 (4)	50		100		50	
Lateral site							
Yes	45 (82)	51	0.99	65	0.75	49	0.43
No	10 (18)	50		78		40	
Cranial site							
Yes	52 (95)	50	0.28	69	0.52	49	0.85
No	3 (5)	50		50		0	
Caudal site							
Yes	31 (56)	35	0.01	56	0.31	39	0.32
No	14 (44)	69		77		56	
Anterior site							
Yes	48 (87)	46	0.11	66	0.38	45	0.86
No	7 (13)	83				57	
Posterior site (pterygoid plates)							
Yes	34 (62)	36	0.01	62	0.41	37	0.29
No	21 (38)	71		73		60	
Nasopharynx							
Yes	5 (9)	60	0.62	65	0.28	47	0.54
No	50 (91)	50		100		40	
Posterior wall^+^							
Yes	50 (91)	55	1	65	0.93	45	0.9
No	5 (9)	60		80		60

**Table 3:** The 5-year OS, LC, and DMFS rates for cN0 cases for each patient characteristic

Characteristic		5-year OS rate (%)		5-year LC rate		5-year DMFS rate	
	No. (%)		*p* value		*p* value		*p* value
Age (years)							
≥ 70 years	23 (61)	47	0.14	78	0.39	48	0.31
< 70 years	15 (39)	58		54		64	
Male	32 (84)	48	0.7	67	0.61	50	0.19
Female	6 (16)	67		67		83	
ECOG PS							
0	29 (76)	55	0.08	67	1	56	< 0.01
1	8 (21)	47		69		28	
≥ 2	1 (3)	–		–		–	
T factor							
3	5 (13)	60	0.4	80	0.31	80	0.78
4a	15 (39)	40		51		45	
4b	18 (48)	60		77		57	
Direction of tumor invasion medial site							
Yes	37 (97)	50	0.78	66	0.53	56	0.5
No	1 (3)	100		100		–	
Lateral site							
Yes	30 (79)	52	0.81	62	0.28	56	0.47
No	8 (21)	50		86		50	
Cranial site							
Yes	37 (97)	50	0.42	66	0.53	50	0.44
No	1 (3)	–		–		–	
Caudal site							
Yes	21 (55)	35	0.04	60	0.35	42	0.27
No	17 (45)	70		74		70	
Anterior site							
Yes	33 (87)	47	0.2	67	0.58	54	0.94
No	5 (13)	80		75		60	
Posterior site (pterygoid plates)							
Yes	20 (53)	37	0.04	67	0.68	43	0.37
No	18 (47)	66		68		66	
Nasopharynx							
Yes	2 (5)	50	0.6	100	0.52	50	0.79
No	36 (95)	51		66		55	
Posterior wall^+^							
Yes	33 (85)	50	0.8	65	0.84	54	0.96
No	5 (15)	60		80		60	

**Fig. 3:**
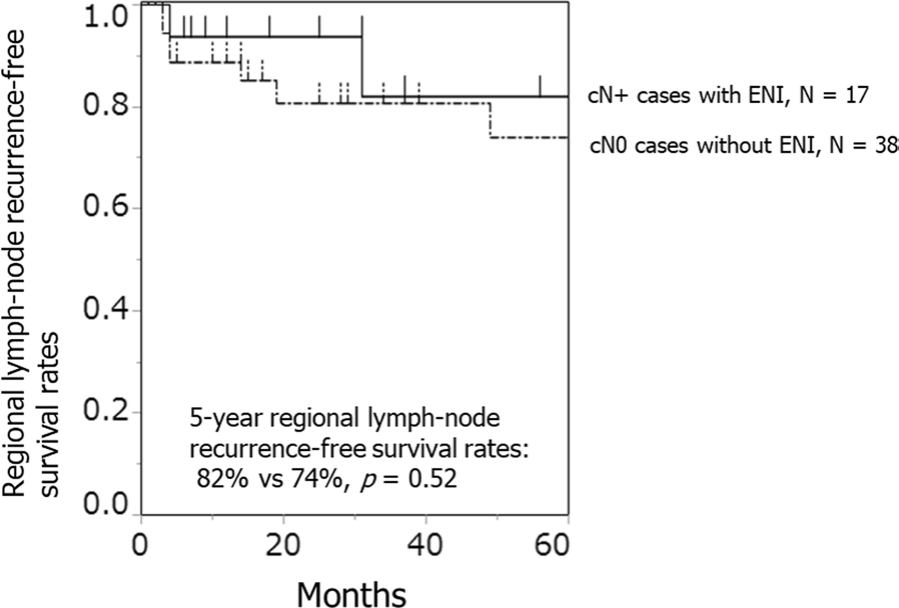
Regional recurrence-free survival rates according to lymph node (LN) status upon commencement of treatment

In the nine cases of regional failure after treatment (median, 4 months; range, 3–49 months), seven cases (18%) were cN0, and two (12%) were cN+. Of the nine regional failures, one was local and regional, and eight were regional only (three occurred after local failure). Salvage surgery was performed in eight of nine cases; one died from surgical complications (brain abscess), five died because of disease progression, and two survived. Figure 2b, c show the distribution of regional failure in cN0 and cN+ cases. Figure 3 demonstrates the regional recurrence-free survival rates according to LN status at the start of treatment.

### Risk factors for regional failure in cN0 cases

For 38 patients with cN0, we analyzed the factors associated with regional failure, which was seen in 7 cases. In the univariate analysis, posterior (*p* < 0.01) and caudal extension (*p* = 0.02) were significantly correlated with the 5-year regional recurrence-free rates (Table 1). Other clinical factors, namely age, PS, and T-stage, showed no correlations. The multivariate analysis showed a significant correlation between posterior extension and regional failure (hazard ratio, 10.5; *p* < 0.01).

### Risk factors for LN metastasis at diagnosis

Table 1 demonstrates the frequency of LN metastasis at diagnosis classified according to patients’ clinical and anatomical characteristics. In the univariate analysis, posterior extension was significantly associated with LN metastasis (41% vs. 13%, respectively; *p* = 0.02) but not with other factors.

### Adverse events

Table 4 summarizes the AEs associated with external radiotherapy and IA chemotherapy. In the non-ENI group, one patient suffered an early death 8 days after the end of treatment because of aspiration pneumonitis, which appeared to be due to treatment-related mucositis and dysphagia. There were no significant differences between the two groups in the incidence of acute high-grade (3 or 4) AEs. There were significantly more cases of high-grade late AEs in the ENI group than in the non-ENI group (41% vs. 13%, respectively; *p* = 0.03).

**Table 4:** Comparison of adverse events between the elective nodal irradiation (ENI) and non-ENI groups

CTCAE v4.0	Non-ENI group (n = 38)				ENI group (n = 17)				p-value
	Grade 1/2 no. (%)	Grade 3 no. (%)	Grade 4 no. (%)	Grade 5 no. (%)	Grade 1/2 no. (%)	Grade 3 no. (%)	Grade 4 no. (%)	Grade 5 no. (%)	
Acute toxicities									
All toxicities ≥ Grade 3		23 (61)				9 (53)			0.8
Mucositis, oral	24 (63)	12 (32)	–	–	11 (65)	5 (29)	–	–	1
Cheilitis	3 (8)	–	–	–	–	–	–	–	
Radiation-induced dermatitis	21 (55)	2 (4)	–	–	5 (29)	1 (6)	–	–	1
Conjunctivitis	13 (34)	–	–	–	5 (29)	–	–	–	
Keratitis	3 (8)	1 (3)	–	–	1 (6)	–	–	–	1
Nausea	4 (11)	–	–	–	–	1 (6)	–	–	1
Anorexia	–	3 (8)	–	–	1 (6)	–	–	–	0.5
Pneumonitis	–	2 (5)	1 (3)	–	–	1 (6)	–	–	1
Depression	–	1 (3)	–	–	–	–	–	–	1
Injury to the carotid artery	–	–	–	–	–	–	1 (6)	–	1
Transient ischemic attacks	–	1 (3)	–	–	–	–	–	–	1
Late toxicities									
All toxicities > Grade 3		5 (13)				7 (41)			0.03
Epiphora	5 (13)	–	–	–	3 (18)	–	–	–	
Dry eye	2 (5)	–	–	–	–	–	–	–	
Cataract	2 (6)	2 (5)	–	–	–	1 (6)	–	–	1
Optic nerve disorders	–	–	2 (5)	–	–	–	–	–	1
Dysesthesia	6 (16)	–	–	–	2 (12)	–	–	–	
Trismus	–	1 (3)	–	–	–	2 (12)	–	–	0.2
Osteonecrosis of the jaw	–	–	–	–	1 (6)	1 (6)	–	–	0.3
Anorexia	–	–	–	–	–	1 (6)	–	–	0.3
Pneumonitis	–	–	–	–	–	2 (12)	–	–	0.09
Stroke	1 (3)	–	–	–	–	–	–	–	

In 11 cases in the IMRT group (ENI = 5, non-ENI = 6), there were 7 acute and 4 late high-grade AEs. Three of the late high-grade AEs were in the ENI group, and all affected patients received 70-Gy radiation doses.

## Discussion

In this study, we treated patients with locally advanced MSC using external beam irradiation combined with superselective IA chemotherapy, and the 5-year LC and OS were 67% and 50%, respectively. Our results were similar to those of other studies [5, 6]. The combination of IA chemotherapy and external radiation therapy or proton therapy can improve LC and OS [7, 13]. Homma et al. reported good results for 5-year LC (66%) and 5-year OS for stage cT4a (67%) and cT4b (57%) after external radiotherapy and IA chemotherapy [6]. Zenda et al. reported a 1-year LC of 77% and 3- and 5-year OS rates of 59% and 55%, respectively, for unresectable paranasal sinus and nasal cavity cancers after proton therapy [13]. ENI was not conducted in these studies, including ours, for cN0 cases.

Two of seventeen patients with cN+ who received ENI in this study developed regional node failure. This incidence was similar to that of patients with cN0, who did not receive ENI. In addition, the 5-year DMFS and 5-year OS rates in both groups were similar (Table 3). However, there were more patients with high-grade late AEs in the cN+ and ENI group than in the cN0 and non-ENI group (41% vs. 13%, respectively; *p* = 0.03). Feng et al. reported grade 3 dysphagia in 8% of cases and aspiration on videofluoroscopy in 44% of cases 3 months after irradiation with a high radiation dose (mean, 64 Gy) to the pharyngeal constrictor muscle [14]. If ENI is performed for all patients with T3 and T4 disease, the incidence of late high-grade AEs may increase with prolonged survival [15]. Therefore, it is necessary to select appropriate candidates for ENI to maximize the clinical benefit in locally advanced MSC.

We divided tumor invasion into six directions, and found that only posterior (to the pterygoid plates) and caudal (to the oral cavity) extension correlated significantly with regional failure. Jeon et al. divided extra-maxillary sinus involvement of the tumor into four directions, and reported that destruction of the posterior wall was a significant risk factor for regional failure in cN0 MSC [11]. We attempted to analyze the relationship between the incidence of regional recurrence and the direction of the tumor invasion according to the classification by Jeon et al. We identified 33 patients with destruction of the posterior wall; however, this was not a significant predictive factor (*p* = 0.3). The categories of destructions of the posterior wall in the study by Jeon et al. included a wide range of extensions (infratemporal fossa involvement), as well as T3 and T4 tumors in the UICC classification [8]. Therefore, we divided the classification of the posterior wall destruction by Jeon et al. into two categories: lateral extension (to the masticator space) and posterior extension (to the pterygoid plates) to identify more appropriate risk factors for regional failure. Extension to the pterygoid plates falls into the T4a category in the UICC classification [8]. Other studies reported that ENI for cN0 cases is necessary only if the tumor extends to a contiguous area, such as the nasopharynx or oral cavity, where lymphatic flow is rich [9, 10]. In the studies, regional failure occurred in only T4b cases of nasopharyngeal invasion, and only two cases showed nasopharyngeal extension in our series. All nasopharyngeal invasions were included in the classification of posterior extension, in our study, because tumors reach this region via the pterygoid plates. In this study, the incidences of posterior wall destruction, invasion of the pterygoid plates, and nasopharyngeal invasion were 87%, 53%, and 5% in 33, 20, and 2 of 38 cN0 cases, respectively. Additionally, our patients had advanced MSC (91% of patients were cT4), and most had destruction of the posterior wall. Therefore, invasion of the pterygoid plates and oral cavity were considered more appropriate risk factors for regional recurrence. We were able to identify patients with a higher risk of regional recurrence more precisely using our new categorization of tumor extension. Regional failure affects distant metastases and OS [4, 11, 16, 17]. We did not perform ENI for cN0 patients, so its usefulness is still unknown. However, according to our analysis of the risk factors for regional recurrence and OS in cN0 cases (Table 3), these patients were considered good candidates for ENI.

To confirm our results, we also examined the risk factors for LN metastases present at diagnosis in the cN+ group. Invasion of the pterygoid plates was the only factor correlated with LN metastases, which supported the results in the cN0 cases. In a multi-center retrospective study by Homma et al., the incidence of LN metastases was significantly higher in cases of nasopharyngeal or oral invasion at presentation among 128 cases of T4 MSC [18]. In all cases of nasopharyngeal invasion, invasion of the pterygoid plates was also observed, which is consistent with our results. Other factors, such as T-stage, PS, or age, showed no association with regional failure.

Regional failure occurred mainly at levels I and II in this study, as reported previously [1]. However, recurrence was also observed in level III in both cN0 and cN+ cases. This may be because of differences between IA chemotherapy and systemic chemotherapy. IA chemotherapy is effective only in the arterial perfusion territory; therefore, this method cannot eradicate tumor cells located in the regions far from the primary tumor. Although contralateral neck irradiation for ENI is recommended in the guidelines [3], this approach is controversial. In our study, we encountered very few contralateral regional failures with or without ENI, despite the finding that the majority of these cases were advanced, with extension into midline structures, namely the oral cavity, nasopharynx, and nasal cavity. These results may obviate the need for contralateral neck irradiation for ENI.

The median irradiation dose to the primary site and LN metastases in our study was 60 Gy, which is lower than the general definitive irradiation dose reported in other studies [3, 5, 6]. The American College of Radiology (ACR) guidelines recommend a definitive irradiation dose of at least 66 Gy [3]. The irradiation dose was reported as 65 Gy in 26 fractions or 70 Gy in 35 fractions in previous studies, even in external beam radiotherapy and IA chemotherapy [5, 6]. We previously reported the treatment results of the combination of superselective IA chemotherapy and radiation therapy, and the total dose of 60 Gy was effective and safe in that setting. Therefore, we treated the patients in this report using the same radiation dose [7]. Previous studies reported high frequencies of the high-grade late adverse event of optic neuropathy (35%) when combining irradiation therapy with IA chemotherapy. Compared with these results, the frequency in our study was relatively low (2/55), and this may be due to the lower radiation dose. Further evidence of the usefulness and optimal procedure for IA chemotherapy will be obtained by an ongoing Phase III clinical trial (University Hospital Medical Information Network (UMIN) Clinical Trials Registry number: UMIN000013706).

The current study has several limitations associated with its retrospective design. First, the diagnostic imaging modalities performed before and after treatment were not standardized. The role of MRI and PET-CT is very important for head and neck tumors [19]. In the early phase of this study, treatment was sometimes started without evaluation by MRI (24/55) or PET-CT (42/55) because of the limited availability of these imaging modalities. Second, external beam radiotherapy was provided using mainly 3DCRT (44/55). Our results, which showed that severe late AEs increased in the ENI group, may have been influenced by the external irradiation technique [20]. Some researchers have reported that IMRT may decrease the number of these AEs [3]. The number of IMRT cases was small in this study, and the relationship between ENI and severe late AEs is unclear. Third, when regional failure was suspected, pathological examination was not performed in all cases; 3/9 cases were diagnosed according to imaging findings. Finally, differences between IA and intravenous chemotherapy might have affected regional control.

In conclusion, ENI for advanced MSC increased the incidence of severe late toxicities. Invasion of the pterygoid plates and oral cavity were high-risk factors for regional failure in cN0 cases, and these patients may be suitable candidates for ENI.

## Data Availability

The data that support the findings of this study are available on request from the corresponding author. Code availability. Not applicable.

## Abbreviations

MSC: Maxillary sinus cancer
ENI: Elective nodal irradiation
cN+: Clinical node-positive
cN0: Clinical node-negative
NCCN: National Comprehensive Cancer Network
IA: Intraarterial
UICC: Union for International Cancer Control
CT: Computed tomography
MRI: Magnetic resonance imaging
PET: Positron emission tomography
3DCRT: Three-dimensional conformal radiotherapy
IMRT: Intensity-modulated radiation therapy
LN: Lymph node
OS: Overall survival
PS: Performance status
AEs: Adverse events
LC: Local control
DMFS: Distant metastatic recurrence-free survival
ACR: American College of Radiology
UMIN: University Hospital Medical Information Network

## References

[1] Abu-Ghanem S, Horowitz G, Abergel A, Yehuda M, Gutfeld O, Carmel NN, Fliss DM (2015). Elective neck irradiation versus observation in squamous cell carcinoma of the maxillary sinus with N0 neck: a meta-analysis and review of the literature. Head Neck.

[2] National Comprehensive Cancer Network (2019) NCCN, Clinical Practice Guidelines in Oncology Head and Neck Cancers, version 3. https://www.nccn.org/professionals/physician_gls/pdf/head-and-neck.pdf. Accessed 21 July 2020. 10.6004/jnccn.2020.0031 32634781

[3] Siddiqui F, Smith RV, Yom SS, Beitler JJ, Busse PM, Cooper JS, Hanna EY, Jones CU, Koyfman SA, Quon H, Ridge JA, Saba NF, Worden F, Yao M, Salama JK, Expert Panel on Radiation Oncology–Head and Neck Cancer (2017). ACR appropriateness criteria^®^ nasal cavity and paranasal sinus cancers. Head Neck.

[4] Bhattacharyya N (2003). Factors affecting survival in maxillary sinus cancer. J Oral Maxillofac Surg.

[5] Homma A, Oridate N, Suzuki F, Taki S, Asano T, Yoshida D, Onimaru R, Nishioka T, Shirato H, Fukuda S (2009). Superselective high-dose cisplatin infusion with concomitant radiotherapy in patients with advanced cancer of the nasal cavity and paranasal sinuses: a single institution experience. Cancer.

[6] Homma A, Sakashita T, Yoshida D, Onimaru R, Tsuchiya K, Suzuki F, Yasuda K, Hatakeyama H, Furusawa J, Mizumachi T, Kano S, Inamura N, Taki S, Shirato H, Fukuda S (2013). Superselective intraarterial cisplatin infusion and concomitant radiotherapy for maxillary sinus cancer. Br J Cancer.

[7] Yokoyama J, Ohba S, Fujimaki M, Anzai T, Kojima M, Ikeda K, Suzuki M, Yoshimoto H, Inoue K (2014). Impact of intraarterial chemotherapy including internal carotid artery for advanced paranasal sinus cancers involving the skull base. Br J Cancer.

[8] Sobin LH, Wittekind CH, Gospodarowicz M (2009). TNM Classification of Malignant Tumours, UICC.

[9] Giri SP, Reddy EK, Gemer LS, Krishnan L, Smalley SR, Evans RG (1992). Management of advanced squamous cell carcinomas of the maxillary sinus. Cancer.

[10] Pezner RD, Moss WT, Tong D, Blasko JC, Griffin TW (1979). Cervical lymph node metastases in patients with squamous cell carcinoma of the maxillary antrum: the role of elective irradiation of the clinically negative neck. Int J Radiat Oncol Biol Phys.

[11] Jeon SH, Han DH, Won TB, Keam B, Kim JH, Wu HG (2017). Implication of tumor location for lymph node metastasis in maxillary sinus carcinoma: indications for elective neck treatment. J Oral Maxillofac Surg.

[12] National Cancer Institute (U.S.) (2009). Common terminology criteria for adverse events (CTCAE).

[13] Zenda S, Kohno R, Kawashima M, Arahira S, Nishio T, Tahara M, Hayashi R, Kishimoto S, Ogino T (2011). Proton beam therapy for unresectable malignancies of the nasal cavity and paranasal sinuses. Int J Radiat Oncol Biol Phys.

[14] Feng FY, Kim HM, Lyden TH, Haxer MJ, Feng M, Worden FP, Chepeha DB, Eisbruch A (2007). Intensity-modulated radiotherapy of head and neck cancer aiming to reduce dysphagia: early dose-effect relationships for the swallowing structures. Int J Radiat Oncol Biol Phys.

[15] Nevens D, Duprez F, Daisne JF, Dok R, Belmans A, Voordeckers M, Van den Weyngaert D, De Neve W, Nuyts S (2017). Reduction of the dose of radiotherapy to the elective neck in head and neck squamous cell carcinoma; a randomized clinical trial. Effect on late toxicity and tumor control. Radiother Oncol.

[16] Carrillo JF, Guemes A, Ramirez-Ortega MC, Onate-Ocana LF (2005). Prognostic factors in maxillary sinus and nasal cavity carcinoma. Eur J Surg Oncol.

[17] Cantu G, Bimbi G, Miceli R, Mariani L, Colombo S, Riccio S, Squadrelli M, Battisti A, Pompilio M, Rossi M (2008). Lymph node metastases in malignant tumors of the paranasal sinuses: prognostic value and treatment. Arch Otolaryngol Head Neck Surg.

[18] Homma A, Hayashi R, Matsuura K, Kato K, Kawabata K, Monden N, Hasegawa Y, Onitsuka T, Fujimoto Y, Iwae S, Okami K, Matsuzuka T, Yoshino K, Nibu K, Kato T, Nishino H, Asakage T, Ota I, Kitamura M, Kubota A, Ueda T, Ikebuchi K, Watanabe A, Fujii M (2014). Lymph node metastasis in t4 maxillary sinus squamous cell carcinoma: incidence and treatment outcome. Ann Surg Oncol.

[19] Pierpaolo A, Riccardo L, Isacco D, Agostino C, Paolo B, Natale Q, Michele F, Evangelistai L, Lorenza M, Federico C, Carmelo T, Paola M, Valentina L, Salvatore A, Maria R, Elisa C, Alba F (2019). Positron emission tomography with computed tomography imaging (PET/CT) for the radiotherapy planning definition of the biological target volume: part 1. Crit Rev Oncol Hematol.

[20] Annamaria D, Valentina L, Claudio Z, Bianca MP, Alessandro F, Martina I, Anna C, Simonetta S, Cynthia A (2018). Is volumetric modulated arc therapy with constant dose rate a valid option in radiation therapy for head and neck cancer patients?. Rep Pract Oncol Radiother.

